# Photosensitizing nanoparticles and the modulation of ROS generation

**DOI:** 10.3389/fchem.2015.00033

**Published:** 2015-05-27

**Authors:** Dayane B. Tada, Mauricio S. Baptista

**Affiliations:** ^1^Departamento de Ciência e Tecnologia, Instituto de Ciência e Tecnologia, Universidade Federal de São PauloSão José dos Campos, Brazil; ^2^Departamento de Bioquímica, Instituto de Química, Universidade de São PauloSão Paulo, Brazil

**Keywords:** ROS, photosensitizer, nanoparticles, Photodynamic Therapy, photoconversion, analytical methods, ROS generation, *in vitro* assays

## Abstract

The association of PhotoSensitizer (PS) molecules with nanoparticles (NPs) forming photosensitizing NPs, has emerged as a therapeutic strategy to improve PS tumor targeting, to protect PS from deactivation reactions and to enhance both PS solubility and circulation time. Since association with NPs usually alters PS photophysical and photochemical properties, photosensitizing NPs are an important tool to modulate ROS generation. Depending on the design of the photosensitizing NP, i.e., type of PS, the NP material and the method applied for the construction of the photosensitizing NP, the deactivation routes of the excited state can be controlled, allowing the generation of either singlet oxygen or other reactive oxygen species (ROS). Controlling the type of generated ROS is desirable not only in biomedical applications, as in Photodynamic Therapy where the type of ROS affects therapeutic efficiency, but also in other technological relevant fields like energy conversion, where the electron and energy transfer processes are necessary to increase the efficiency of photoconversion cells. The current review highlights some of the recent developments in the design of Photosensitizing NPs aimed at modulating the primary photochemical events after light absorption.

## 1. Introduction

Reactive Oxygen Species (ROS) are reactive molecules derived from molecular oxygen. Superoxide anion, hydrogen peroxide and other peroxides, hydroxyl radical, and singlet oxygen (^1^O_2_) are the main examples of ROS. They are formed both in physiological and pathological conditions and are the main species generated during the photodynamic reactions (Wang and Yi, 2008; Dewaele et al., 2010). Although ROS are usually reported as toxic species due to their high reactivity, which causes several deleterious events, ROS also play key roles as intra/inter-cellular messengers in normal cell signal transduction and cell cycling (Wu, 2006; Park et al., 2011).

Some biomedical therapies make use of ROS in order to kill abnormal cells or microorganisms. One of these therapies is the Photodynamic Therapy (PDT), a well-known method to treat cancer and other diseases by causing cell death through photo-oxidation of biomolecules. Photodynamic reactions occur in the presence of three main components: photosensitizer (PS), light and oxygen (Macdonald and Dougherty, 2001; Oleinick et al., 2002; Brown et al., 2004; Castano et al., 2004; Hamblin and Hasan, 2004). PDT requires loading the cellular tissue with a photosensitizer (PS). The PS molecules are able to transfer energy from their excited state (produced by light absorption) to molecular oxygen, generating ROS. These photosensitized oxidation reactions are also the main actors during exposition of human skin to sun light in the UVA and visible regions (Herrling et al., 2006; Chen and Wang, 2012; Chiarelli-Neto et al., 2014).

**Figure 1:**
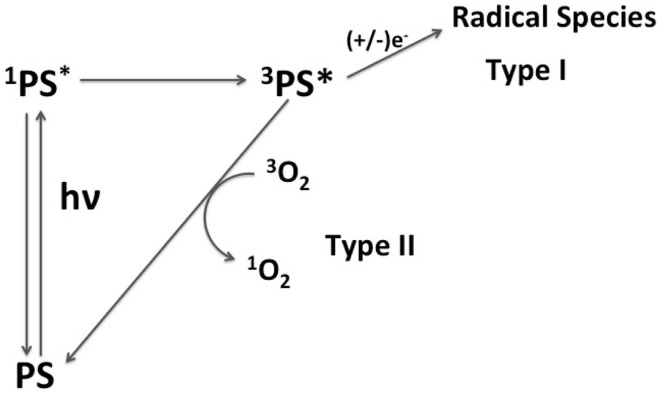
**Scheme of mechanisms of action of a PS**. Mechanism type I is characterized by the reduction/oxydation of triplet excited state of PS (^3^PS^*^) by biomolecules, forming radical species. In the mechanism type II, ^3^PS^*^ transfers energy to molecular oxygen generating ^1^O_2_.

A PS may act through two main mechanisms, i.e., type I and type II (Figure 1). In type I, the excited triplet state of PS reacts with biomolecules forming radical species that may further react with oxygen to form other ROS. In type II, the excited state triplet of PS reacts directly with oxygen, generating ^1^O_2_. Both mechanisms lead to cell death. However, mechanisms type I and type II cause different types of damage (Kochevar et al., 2000; Oleinick et al., 2002). For example, Kochevar and co-authors reported that type I photoreactions are better correlated to cell death through necrosis whereas type II has better correlation to cell death through apoptosis (Kochevar et al., 2000). Apoptosis is a programmed and more controlled mechanism of cell death, which avoids unspecific inflammatory responses after PDT treatment. Therefore, large efforts are being made to allow direct control of PS activity by favoring either type I or type II mechanisms (Morgan and Oseroff, 2001; Almeida et al., 2004; Hilf, 2007; Deda et al., 2013).

Although encouraging results are always reported for *in vitro* assays of PDT, clinical trials have shown limited therapeutic efficiency mainly due to PS low solubility in aqueous media, which leads to PS aggregation and low ^1^O_2_ generation. Other limitations are: poor PS selectivity to accumulate into target tissues; unspecific photodynamic effect, and consequently, damage in healthy cells and prolonged photosensitivity in patients; and negative interference of molecules from the biological tissues on the photophysical and photochemical properties of the PS (Konan et al., 2002; Brown et al., 2004; Detty et al., 2004; Yao et al., 2014). In order to overcome these difficulties, PS association to nanoparticles (NPs), resulting in photosensitizing NPs, has emerged as a successful strategy. Photosensitizing NPs can improve PS solubility, enhance PS circulation time and protect PS from deactivation reactions. NPs also improve tumor targeting, due to the enhanced penetration and retention effect (EPR effect) that 50–100 nm particles exhibit to tumors (Faraji and Wipf, 2009; Doane and Burda, 2012). Besides this passive mechanism of tumor targeting, NPs can also be accumulated in tumors by active targeting mechanisms. This process is based on the specific binding between a ligand, present on the NPs surface, and a receptor from the cytoplasmic membrane of target cells (Konan et al., 2002; Kumar et al., 2008; Hah et al., 2011). NP surface is functionalized with molecules able to recognize receptors super expressed specifically by cancer cells. Targeting ability of NPs can go even further, enhancing PS accumulation in specific subcellular compartments. Control of PS intracellular fate can be achieved by selecting NP material, size, superficial charge and by modifying NP surface with membranes and targeting motif (Morgan and Oseroff, 2001; Tang et al., 2005; Tada et al., 2014). Targeting key organelles like lysosomes and mitochondria induced cell death trough apoptosis rather than necrosis (Ichinose et al., 2006; Deda et al., 2013). These findings provided a new branch of development of PDT drugs with driven-cytolocalization and specific mechanisms of programmed cell death.

Once photosensitizing NPs reach the target tissue and the subcellular compartment, a slow kinetic process can release PS, or it can remain associated with the NP matrix. In this case, it is expected that the whole system (PS+NP) act as a unique PDT agent showing better therapeutic efficiency compared to PS alone.

Additionally to the advantages described above, photosensitizing NPs may be designed to modulate ROS generation. Controlling the amount and type of photoinduced ROS is desirable not only in biomedical applications, but also in other technological relevant fields like energy conversion, where the electron and energy transfer processes are necessary to increase the efficiency of photoconversion cells.

Several NP structures have been designed in order to enhance ROS generation like self-assembled PS, self-assembled PS-DNA conjugate, nanocrystals and carbon dots (Baba et al., 2007; Christensen et al., 2011; An et al., 2012; Ghosh et al., 2014; Nogueira et al., 2014). Although these papers did not report amounts of photoinduced ROS, they reported improved photoactivity, which is possibly a result of the enhanced ROS generation. However, they were not included here because they flee from the focus of this review.

The present work addresses only systems prepared by the association of a PS to a NP matrix, which we call here as photosensitizing NPs. There are many ways through which ROS generation can be modulated by photosensitizing NPs, for example, by controlling PS aggregation and by protecting the PS from deactivation by biomolecules. Some of the recently developed photosensitizing NPs, which were designed to modulate ROS generation are described regarding their mechanism of modulation, photophysical/photochemical properties and final photoactivity. Furthermore, in the end of the chapter we provided experimental details of the methods that were developed and tested to synthesize photosensitizing NPs, to quantify ROS generation and to test photoactivity.

## 2. Photosensitizing NPS with controlled PS aggregation

Most PS molecules aggregate easily in aqueous media. Self-aggregated states reduce fluorescence quantum yields, triplet states and ^1^O_2_ generation, thereby reducing photoactivity (Severino et al., 2003; Gabrielli et al., 2004; Tardivo et al., 2005). While PS aggregation reduces ^1^O_2_ generation, it can improve generation of radical species, for example hydroxyl and peroxyl radicals (Severino et al., 2003; Tardivo et al., 2005; Yoon et al., 2014). Several recent papers have reported on different approaches in order to control PS aggregation. New PS molecules less prone to aggregate were designed with chemical groups that have weak intermolecular interactions or that are bulkier avoiding aggregation by steric effect (Uchoa et al., 2011; de Assis et al., 2013; dos Santos et al., 2013). However, once in the biological media, interactions of PS with biomolecules or with membranes may alter the monomer/aggregate equilibrium (Junqueira et al., 2002; Severino et al., 2003; Gabrielli et al., 2004).

**Figure 2:**
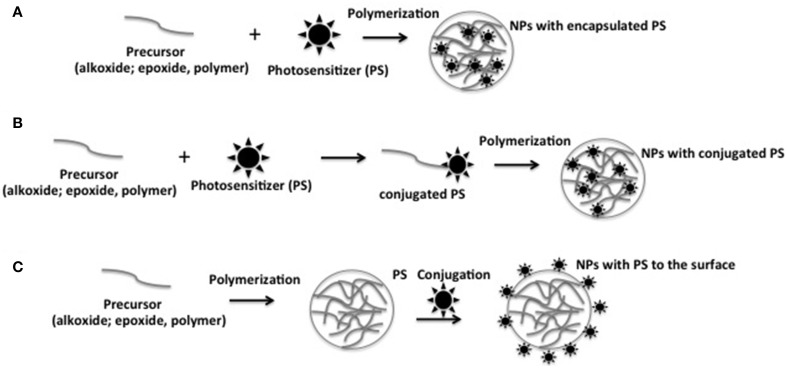
**Schematic representation of the different approaches to prepare photosensitizing NPs. (A)** Encapsulating PS during NP synthesis by physic-sorption; **(B)** chemical conjugation of the PS to NP matrix; **(C)** chemical conjugation of the PS to NP surface.

Photosensitizing NPs have emerged as an alternative tool to control PS aggregation in aqueous or in biological medium. By selecting appropriated NP matrix, PS type and loading method, one can control the ratio of monomer to aggregated PS into the photosensitizing NPs. Because PSs are protected from the direct contact with the biomolecules present in the vicinity, their photophysical properties are also preserved from external interferences (Tada et al., 2007; Rossi et al., 2008; Tada et al., 2010; Yoon et al., 2014). In generic terms, photosensitizing NPs have been prepared by: (i) encapsulating PS during NP synthesis by physic-sorption, and (ii) chemically conjugating the PS to NP matrix or to NP surface (Figure 2).

Two different research groups, Baptista's and Kopelman's groups, have proposed methods to control ROS generation by using photosensitizing NPs containing the phenothiazine dyes, i.e., thionin (Th) and methylene blue (MB). Th and MB are low cost PSs whose widespread use in PDT has been hampered due to their susceptibility to aggregate and the tendency to get reduced in the presence of common biological reducing agents such as NADH. It has been shown that interaction with membranes and interfaces induces aggregation of MB, favoring electron transfer reactions within the dimer, changing the main photochemical mechanism from the generation of ^1^O_2_ to the generation of radical species (Junqueira et al., 2002; Severino et al., 2003; Tardivo et al., 2005).

Baptista's group developed photosensitizing NPs containing Th and MB at different ratios of dimer to monomer into silica NPs (Tada et al., 2010). They compared quantum yield of ^1^O_2_ generation (S) of photosensitizing NPs prepared by PS encapsulation into silica NPs and by PS conjugation to the silica NP surface. ^1^O_2_ generation was quantified by direct measurement of ^1^O_2_ phosphorescence decay and by indirect method, oxidation of the probe 1,3-diphenylisobenzofuran. Higher S was obtained by NPs with lower dimer to monomer (D/M) ratios of PS molecules (Table 1). Photosensitizing NP containing Th encapsulated presented an *S*-value 10 times and 20 times higher than NPs containing MB encapsulated and Th conjugated at NP surface, respectively. The highest S of NPs with encapsulated Th was explained by the lowest D/M ratio of Th in this NP compared to the others. It is important to emphasize that the ratio of PS dimer to monomer was not changed by the interaction with model matrix molecules showing that these photosensitizing NPs can be applied to modulate ROS generation in biological medium.

**Table 1:** **Quantum yield of ^1^O_2_ generation (S) of NPs containing MB or Th in different ratios of dimer to monomer (D/M)**.

**Nanoparticle**		**D/M ratio**	**S direct** **(S/S_0_)**
Th-encapsulated	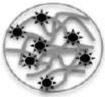	0.0	0.40 (1.00)
Th-conjugated to the surface	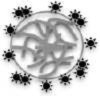	0.8	0.05 (0.10)
MB- encapsulated	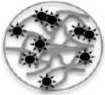	1.6	0.02 (0.05)

**Figure 3:**
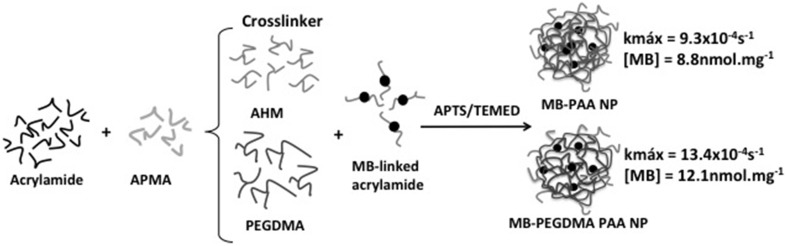
**Preparation of polyacrilamide NPs with encapsulated MB**. The substitution of 3-(acryloyloxy)-2-hydroxypropyl methacrylate (AHM) for a longer crosslinker (poly(ethylene glycol) dimethacrylate; PEGMA) resulted in photosensitizing NPs with larger k. Figure adapted from Hah et al. (2011).

Kopelman's group loaded polyacrylamide NPs with MB by encapsulation and chemical conjugation. MB aggregation was avoided by controlling MB loading. When chemical conjugation approach was used, MB aggregation was controlled by the use of a longer crosslinker in the NP matrix. ROS generation by photosensitizing NP was estimated by a kinetics-based method (see details in analytical methods) where the rate constant (*k*) of decay of fluorescence of anthracene-9,10-dipropionic acid (ADPA) under oxidative quenching by ROS was determined. Upon increasing MB loading, higher *k*-values were observed until it reached an upper limit. Higher MB loading induced MB aggregation causing self-quenching of excited species and ROS inside the NP matrix. This limitation could be overcome by using longer cross-linker in the NP matrix increasing the distance between conjugated MB molecules and reducing probability of collision between excited MB and/or produced ROS (Figure 3). The use of longer crosslinker provided a *k*-value maximum of 13.4 × 10^−4^ s^−1^ at MB loading of 12.1 nmol.mg^−1^. This *k*-value maximum was 1.44 times higher than the value found with photosensitzing NP containing shorter cross-linker, namely *k* = 9.3 × 10^−4^s^−1^ at 8.8 nmol mg^−1^ of MB loading (Hah et al., 2011).

**Figure 4:**
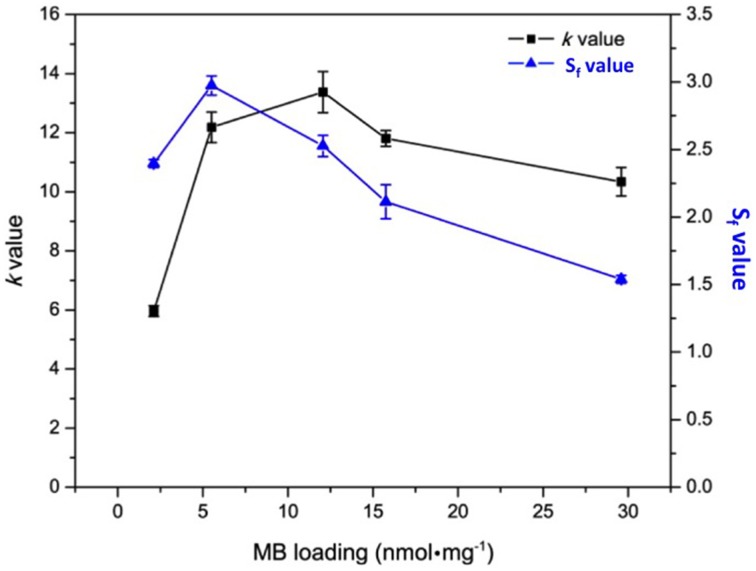
***k*** **and** ***S_f_*****-values of polyacrylamide NPs containing encapsulated MB at different concentration**. Figure adapted from Yoon et al. (2014).

In order to discriminate ^1^O_2_ among all the generated ROS, Kopelman's group used the probe SOSG that emits enhanced fluorescence at 525 nm after reacting with ^1^O_2_. Fluorescence enhancement ratio (Sf) was calculated by the ratio of SOSG fluorescence in the absence and presence of photosensitizing NP. Under irradiation of photosensitizing NP containing longer cross-linker, the value of Sf was higher at MB loading of 5.5 nmol.mg^−1^. Upon increasing MB loading above this concentration, *Sf*-value decreased almost linearly. The lower *Sf*-value at higher MB loading was probably due to MB aggregation that reduced ^1^O_2_ level while ROS level remained high (Figure 4). High ROS generation and low ^1^O_2_ generation is characteristic of MB dimerization (Tardivo et al., 2005).

Comparing encapsulation and conjugation method of preparing of photosensitizing NPs, Kopelman's group concluded that the conjugation method was the most efficient, generating higher PS loading, negligible leaching of MB from the NP and better photoactivity. In fact, photoactivity of NPs prepared by conjugation was 9 times higher than that of NPs with MB encapsulated (Hah et al., 2011).

**Figure 5:**
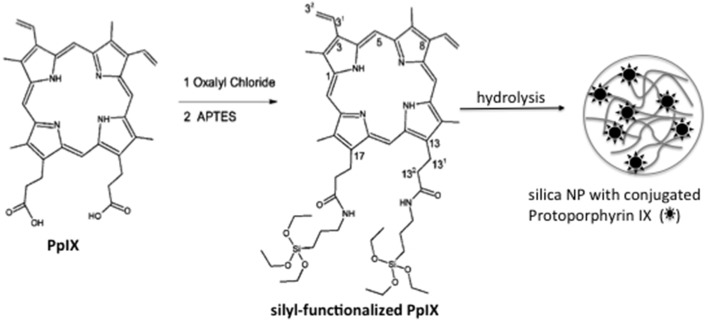
**Schematic representation of photosensitizing NPs synthesis**. Firstly, PpIX was chemically conjugated to the organosilane reagent (APTES). Following, the hydrolysis/conjugation of sylil-PpIX leads to the silica photosensitizing NPs. Figure adapted from Rossi et al. (2008).

Porphyrin is the main lead drug for PDT. Usually the biological photoactivity of porphyrins is hampered by their poor solubility in aqueous medium and high tendency to aggregate. In the work of Rossi et al. (2008), both limitations (solubility and aggregation) were overcome by the entrapment of protophorphyrin IX in silica NPs. In order to improve protoporphyrin loading and to avoid PS leakage from NP matrix, PpIX was chemically conjugated to an organosilane reagent. Photosensitizing NPs were prepared by allowing the silyl-PpIX molecule to participate in the hydrolysis/condensation reactions (Figure 5). ^1^O_2_ generation by photosensitizing NP was indirectly measured using the probe 1,3-diphenylbenzofuran. Compared to the free PpIX, photosensitizing NP presented higher ^1^O_2_ generation efficiency. The increase in the efficiency of ^1^O_2_ generation was shown to be a consequence of a decrease in the monomer-dimer equilibrium since the PS is firmly attached to the NP matrix, whereas in solution, dimerization equilibrium takes place decreasing the yield of ^1^O_2_.

## 3. Photosensitizing NPs that protects PS from deactivation

While controlled aggregation is important to modulate which kind of ROS (^1^O_2_ or radical species) is generated, protection of PS molecules from deactivation by biomolecules is essential to ensure ROS generation in the biological tissue being treated. Several authors have shown that some PSs may be reduced by co-enzymes causing the formation of the inactive leuco-form, leading to erratic results in cell cultures and clinical trials (Cincotta et al., 1993; Tardivo et al., 2005; Pelletier et al., 2006; Tada et al., 2010; Oliveira et al., 2011). By comparing properties of PS free in solution to those encapsulated or conjugated to NPs, several studies have demonstrated that when loaded in NPs, PSs are protected from environmental influences in the ground, singlet and triplet excited states (Tang et al., 2005; Rossi et al., 2008; Tada et al., 2010).

Baptista's group demonstrated that when linked to silica NPs, by encapsulation or conjugation to the NP surface, Th and MB are not accessible to reduction by NADPH molecules (Tada et al., 2010). Therefore, NPs are able to inhibit the chemical reduction of phenothiazines in biological environments, which is one of the main restrictions to their application in PDT. Similarly, Tang et al. (2005) showed that NPs matrix of poliacrylamide successfully protected encapsulated MB from reduction by enzymes. Similar results were obtained in the work of Hah et al. where photosensitizing NPs were prepared by conjugation of MB to a long-chain crosslinker (Hah et al., 2011). Although the resultant structure was more porous, MB was still protected from reduction by biomolecules from external medium.

**Figure 6:**
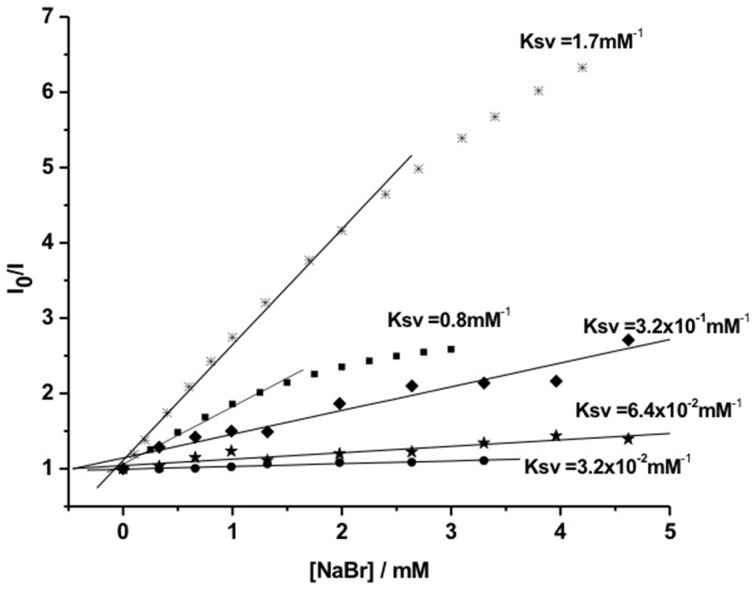
Stern-Volmer curves for the fluorescence quenching process by bromide ions of: Th (^*^) and MB (■) free in solution and photosensitizing NPs. (⋆) Th conjugated to NP surface; (♦) encapsulated Th and (•) encapsulated MB. Figure adapted from Tada et al. (2010).

Protection of singlet-excited states of PSs from deactivation by external quenchers was evaluated by fluorescence quenching assays (Tada et al., 2010). In order to evaluate the kinetics of the photophysical intermolecular deactivation process, Stern-Volmer constants (Ksv) were estimated for the quenching process of free PS and photosensitizing NPs. It was observed that bromide ions were able to quench MB and Th encapsulated and conjugated to the surface of silica NPs. However, quenching constants of photosensitizing NPs were lower (Ksv ca. 6–30 times) than the quenching constants observed for the free PSs in solution (Figure 6). Comparing Ksv values of photosensitizing NPs with encapsulated PSs to Ksv values of NPs with PS conjugated to the surface, it was possible to observe that when PS is present in the NP surface it is more susceptible to quenching. Photosensitizing NP with Th conjugated to the surface showed a value of Ksv 5 times higher than NPs with encapsulated Th.

Additionally, photosensitizing NPs can also avoid deactivation of PS triplet excited states. Assays with sodium ascorbate, an efficient suppressor of MB triplets and a poor suppressor of ^1^O_2_, allowed the comparison between triplet MB deactivation when free in solution and loaded into silica NPs. It was observed that the generation of ^1^O_2_ by MB free in solution was inhibited in the presence of ascorbate, reducing the emission at 1270 nm by more than 99%. In the case of photosensitizing NP containing encapsulated MB, no changes in the emission intensity at 1270 nm were observed after addition of sodium ascorbate, indicating that the deactivation of MB triplets by ascorbate was avoided by silica NP matrix.

The results described above clearly show that photosensitizing NPs are important tools for guarantee ROS generation in biological media, by avoiding PS deactivation by biomolecules. It is important to note that NPs allow the diffusion of ^1^O_2_ from NPs to the external medium, since similar ^1^O_2_ lifetimes were observed when photosensitization occurred both from free PSs and from PS linked to NPs (Tada et al., 2007, 2010; Rossi et al., 2008).

## 4. Modulation of ROS generation by PS association with metal NPs

Metal nanoparticles (NPs) have found applications in different fields such as catalysis, energy, sensors and biomedicine. Biological applications of metal NPs include drug delivery systems for cancer therapy, hyperthermia and agents for diagnosis. Furthermore, metal NPs such as gold, silver and platinum NPs have also been reported to have antibacterial and antifungal properties (Cho et al., 2005; Cheng et al., 2008; Narband et al., 2008; Managa et al., 2014; Zhao et al., 2014). In PDT, metal NPs are being used as PS carrier, coupling hyperthermia, antimicrobial and optical properties of metal matrix to the photodynamic activity of PS (Rossi et al., 2008; Cheng et al., 2011; Khaing Oo et al., 2012; Li et al., 2013; Lkhagvadulam et al., 2013; Fan et al., 2014). Metal-based photosensitizing NPs presented improved phototoxicity against mammalian and microbial cells compared to the therapeutic effect of PS alone. The effects of metal matrix of NP on PS photoactivity vary from system to system. Metal matrix was able to enhance generation of ^1^O_2_ as well as of radical species. In this section, we review some of the metal photosensitizing NPs and discuss how the metal matrix interferes on photophysical/photochemical properties of associated PS.

One of the main features of metal NPs is their strong plasmon field created by surface plasmon resonance. The field intensity decreases with the distance from the metal surface. When a fluorophore or PS is placed at the vicinity of the metal NP (about 10 nm from the metal surface) the electrons of the PS that are involved in the excitation/emission process, interact with the plasmon field of the metal NP (Dulkeith et al., 2002; Schneider et al., 2006). The interaction results in quenching or enhancement of the fluorescence level of PS and consequently of radical species and/or ^1^O_2_.

**Figure 7:**
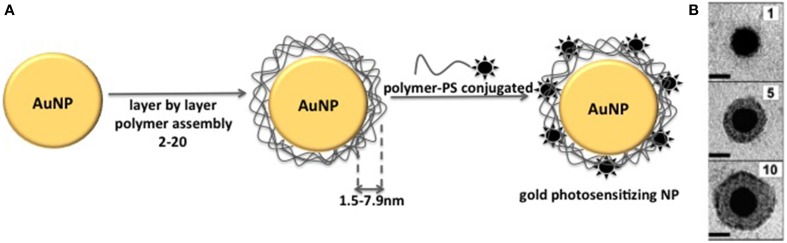
**(A)** Schematic representation of the method used to prepare gold photosensitizing NPs with PS at different distances from NP core. Layer by layer coating of gold NP with polymer leads to coating thickness of 1.5–7.9 nm. The final step is the NP coating with the polymer conjugated to the PS molecule. **(B)** Transmission electron micrographs of individual gold NPs coated with increasing number of polymer layers. Figure adapted from Schneider et al. (2006).

Several studies have been dedicated to the understanding of how plasmon field of metal NP affects fluorescence emission of associated fluorophore (Dulkeith et al., 2002; Samia et al., 2003, 2006; Schneider et al., 2006; Kang et al., 2011; Khaing Oo et al., 2012; Lkhagvadulam et al., 2013). These studies have been performed by using metal NPs of different sizes and fluorophores associated to the NP matrix at different distances (Figure 7). The results showed that the main factors to be considered aiming at the modulation of fluoresecence are: (i) metal composition of NP matrix (Au, Pt, Ag, Cu) (ii) NP size (iii) chemical nature of the fluorophore (excitation/emission spectrum, fluorescence quantum yield) (iv) coating of NP matrix.

Kang et al. (2011) showed the modulation of fluorescence emission of cypate by controlling the distance between the fluorophore and the surface of 10 nm gold NPs. In order to control the distance of cypate from NPs matrix, gold NPs were coated with polymer layers at known thicknesses and cypate was placed outside these layers. They observed that cypate fluorescence became almost completely quenched on the particle surface. However, at 4.5 nm from the gold NP surface, fluorescence was approximately 17 times stronger than that observed for the free cypate. As the distance increased further, the enhancement decreased. Notably, distant-dependent enhanced fluorescence was not observed for all NP-fluorophore systems. For example, Schneider et al. (2006) also controlled the distance between FITC and gold NPs with polymer layers. However in this case, FITC fluoresecence was always quenched, independently on the distance between the fluorophore and the NP surface.

The enhanced/decreased fluorescence of PS when it is associated to metal NPs not only affects PS therapeutic efficiency but also interferes in the fluorescence-based monitoring of PS biodistribution. However, even when the PS is non-fluorescent, its association with metal NPs enables PS monitoring through surface-enhanced Raman spectroscopy (SERS). SERS is another phenomenon provided by metals in which the intensities of Raman signals are increased in several orders of magnitude when the PS is in the proximity of metal surfaces (Faulds et al., 2010; Costa et al., 2011). Based on SERS, recent works have developed photosensitizing NPs composed of metal NPs and non-fluorescent PS with simultaneous photoactivity and imaging features (Tam et al., 2012; Farhadi et al., 2015).

**Figure 8:**
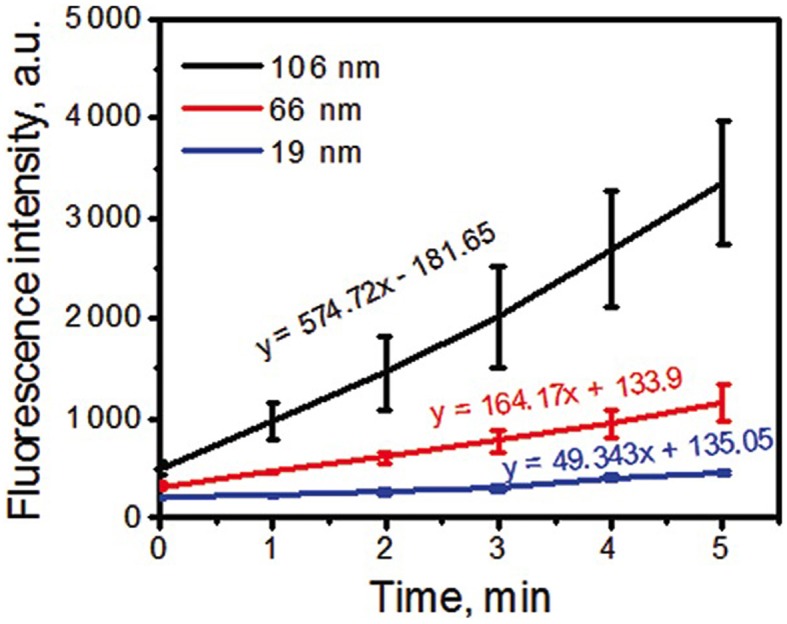
**Kinetics of ROS formation of PpIX-conjugated to AuNPs with tracking agent DHR123**. Larger NPs resulted in higher ROS generation. Figure extracted from Khaing Oo et al. (2012).

Association of a PS to the metal NP can also affect PS efficiency in terms of ROS generation. Both radical species and ^1^O_2_ generation can be significantly enhanced in metal photosensitizing NP. Khaing Oo et al. (2012) have shown that conjugation of protophorphyrin IX on the surface of gold NPs enhanced ROS generation in a size-dependent manner. Photosensitizing NPs of 106 nm of diameter presented an enhancement ratio of 11 and of 3.5 times higher than photosensitizing NPs of 19 and 66 nm, respectively. Theoretical simulations of the electromagnetic field enhancement by gold NPs contributed to confirm that ROS generation is significantly enhanced by localized plasmonic field of gold NPs. The larger the NPs were, the stronger the effect was (Figure 8). Interestingly, when intracellular level of ^1^O_2_ was measured, the highest enhancement was observed in cells that were treated with 66 nm photosensitizing NPs and not with 106 nm NPs. Clearly, intracellular level of ^1^O_2_ is associated with the cell uptake of NPs, which was higher for 66 nm NPs. Also, it is important to note that intracellular measurements were performed with a probe that detects only ^1^O_2_ among all the ROS produced by the photosensitizing NPs. In this way, it is possible that 106 nm NPs enhanced ROS generation but not specifically ^1^O_2_ generation as observed by Yoon et al. (2014) in their studies with polymeric photosensitizing NPs.

**Figure 9:**
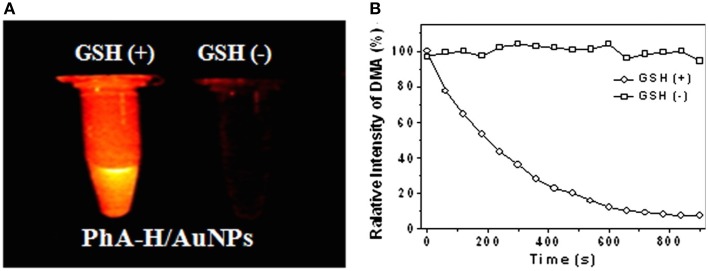
**(A)** Fluorescence images of PhA-gold NPs suspension in the presence and absence of GSH. **(B)** Change in 9,10-dimethylanthracene (DMA) fluorescence in the presence and absence of GSH. DMA fluorescence decrease with the generation of singlet oxygen. Figure extracted from Li et al. (2013).

In some photosensitizing NPs, metal matrix of NP can quench the photoactivity of PS instead of enhancing it. Despite of that, this kind of photosensitizing NP presented potential application in PDT. Li et al. (2013) reported one of these systems. In their work, a photosensitizing NP was designed to be photoinactive in the circulatory system and to release the PS only inside cells. The PS pheophorbide a (PhA) was conjugated to thiolated-heparin molecules. The final conjugated was linked to gold NPs through thiol groups of heparin. PhA was found to be non-fluorescent and photo-inactive when incorporated in the gold NP. However, when glutathione (GSH) was added to PhA-gold NPs suspension, PhA was released from gold NPs recovering its fluorescence and ROS generation (Figure 9). In *in vitro* assays, cellular death was observed after cell incubation with PhA-gold NPs followed by light irradiation. Cellular death was attributed to the PhA release inside the cells due to the high concentration of glutathione, which was able to break gold-thiol linkage. The photo-inactive property of PhA while linked to gold NPs and its triggered release inside cells, make the heparin/PhA-gold NPs very attractive for PDT of cancer since it could favor pharmacokinectics of PhA and also reduce PS side effects.

**Figure 10:**
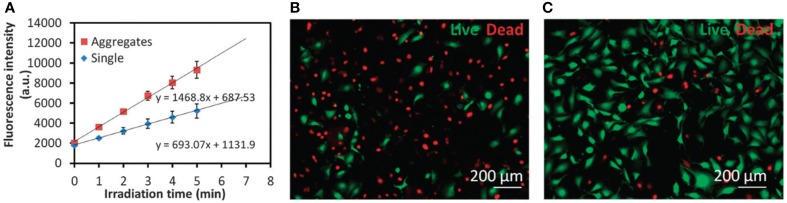
**(A)** Time-resolved kinetics of ROS formation by PpIX in the presence of single Au NPs or Au NP aggregates under a broadband light irradiation for different time intervals. DHR123 was used as the probe to detect ROS. **(B,C)** Representative fluorescence images of MDA-MB-231 cells stained with a Live/Dead kit after PDT treatments with **(B)** a combination of 5-ALA and intracellular induced Au NP aggregates or **(C)** 5-ALA only. Figure extracted from Yang et al. (2014).

Although several reports have shown enhanced ROS generation by chemical conjugation of PS in the monomeric form on the NPs surface, covalent linkage of the PS to NP matrix is not essential to achieve an enhanced photodynamic effect. Recently, Yang et al. (2014) have shown that ROS generation by free PS can be further enhanced in the presence of gold NPs aggregates. It was observed that ROS generation by protophorphyrin IX in the presence of aggregated gold NPs was 2 times higher than ROS generation by the PS in the presence of non-aggregated Au NPs (Figure 10A).

In *in vitro* studies, it was observed that Au NPs aggregation enhances not only ROS levels but also ^1^O_2_ levels. By incubating cells with positively and negatively charged gold NPs, intracellular Au NPs aggregation was promoted (Yang et al., 2014). Elevated levels of ^1^O_2_ generated by the PS 5-ALA inside cells were achieved. The intracellular level of ^1^O_2_ was 4 times higher than the observed for 5-ALA alone. The enhanced generation of ^1^O_2_ resulted in a cellular death 3 times higher than the cellular death observed after cell treatment with 5-ALA alone (Figures 10B,C).

The results obtained by Yang et al. (2014) were explained by the fact that when metallic NPs are brought close, their transition dipoles couple to each other, which are capable of distributing the intensity of localized electromagnetic field, creating regions of highly confined electric field. Due to the coherent interference of enhanced fields at the junction of the particles, larger electromagnetic fields are yielded, which in turn, amplify the energy delivered to the PS. Importantly, due to the high absorbance of gold NPs aggregated in the NIR region, enhanced ROS generation could be observed even upon excitation by longer wavelengths (λ > 600 and λ > 700 nm). This benefits the clinical application of gold NPs aggregates since NIR light can penetrate much deeper in biological tissue (Castano et al., 2004).

Narband et al. (2008) presented another example of photosensitizing NP that did not require covalent bond between PS and NPs matrix. They prepared a photosensitizing NP by promoting adsorption of toluidine blue O (TBO) to gold NPs. The photosensitizing NP presented enhanced TBO photoactivity against Staphylococcus aureus even if significantly less ^1^O_2_ was formed by TBO. The authors suggested that the adsorption of the TBO molecules onto the surface of the gold NPs, through the sulfur atom on the TBO, allowed it to harvest more incident light per molecule, which was proved by the increase in the extinction coefficient of adsorbed TBO. This produced an excited state of the PS, which quickly decayed by some sort of dark process, since no PS-fluorescence was observed. Nonetheless, the non-radiative process did not allowed the formation of ^1^O_2_. Probably, TBO is suppressed by a redox route resulting in the generation of other ROS, possibly hydroxyl radicals, which could explain the increased anti-bacterial photoactivity.

In the work reported by Managa et al. (2014), a metal photosensitizing NP was prepared by conjugation of gallium tetra-(4-Carboxyphenyl) porphyrin with platinum (Pt) NPs. These NPs were embedded in polystyrene fibers. Compared to the polystyrene fiber prepared with free porphyrins, polystyrene fiber containing photosensitizing NPs presented enhanced antibacterial activity. Differently from the works mentioned above, they did not observe an increase of ^1^O_2_ quantum yield or any other ROS in the Pt NPs. In fact, the authors suggested that bacterial inhibition was a result of a synergistic effect of antimicrobial activity of Pt and photoactivity of PS.

Overall, the results mentioned above, showed that metal NPs have a wide application in the development of photosensitizing NPs. Photophysical and photochemical properties of a PS are altered by coupling the PS to metal NPs. As a result, new applications for the photosensitizing NPs have arisen, for example as biomedical devices like drug delivery systems, biosensors or agents for PDT. In PDT, improved photoactivity against mammalian and microbial cells has been reported mainly as a consequence of the modulation of ROS generation by PS coupled to metal NPs.

## 5. Conclusions

In recent years numerous NP structures have been designed for biomedical applications. In cancer therapy, they emerged as a tool to improve tumor targeting and to reduce collateral effects of conventional chemotherapeutic drugs. Until today, two NPs have been approved by FDA for cancer treatment: Doxil and Paclitaxel. Additionally to the improved tumor targeting which leads to the reduced collateral effects, in PDT, NPs have been widely explored due to their ability to enhance ROS generation and to protect PS from deactivation process (reactions with biomolecules or PS aggregation).

Several strategies have been used in order to prepare NPs for PDT applications. Among them, we highlight the association of PS to a NP matrix, resulting in the photosensitizing NP. Different approaches have been used to produce photosensitizing NPs. PS encapsulation/entrapment into NP matrix result in a photosensitizing NP where PS is present mainly inside the NP, from where it can be photoactivated. The PS conjugation to NP surface produce a structure with no PS inside the matrix, only at the surface, facilitating ROS availability to the external medium but also making PS more susceptible to deactivation by biomolecules. A third approach to prepare photosensitizing NPs is the PS conjugation by covalent bond to the precursor of the NP matrix, generating a new molecule of PS-functionalized precursor. Polymerization/hydrolysis reaction of this precursor leads to a photosensitzing NP that contains PS covalently attached into the NP matrix and also on the NP surface. This photosensitizing NP has presented no PS leakage and some advantages in terms of controlling PS loading and distribution.

Photosensitizing NPs are not only important due to their targeting ability but also as a tool for the modulation of ROS generation. Photoinduced generation of ROS can be modulated by selecting the association method, the NP material and the PS itself. This work addressed the potential application of some selected types of photosensitizing NPs as modulators of ROS generation. Three approaches were described: protection of PS from deactivation by external interferences, controlled aggregation of PS and controlled photophysical/photochemical behavior of PS by plasmon fields of metal surfaces. The photosensitizing NPs presented enhanced or reduced generation of ROS compared to PS alone. Additionally, in some cases free-radical generation was enhanced as ^1^O_2_ generation was reduced and vice versa. Enhanced and modulated ROS generation make photosensitizing NPs very attractive systems for biological applications, especially as agents for PDT where enhanced ROS generation improves cell death of microbial and mammalian cells.

We believe that designing of photosensitizing NPs will continue to be an area of highly active research toward the modulation of quantity and type of generated ROS (radical species or ^1^O_2_) in order to control the efficiency and type of photoinduced cell death (apoptosis or necrosis). The development of photosensitizing NPs will be increasingly assisted by computational tools in order to fully understand the consequences of PS attachment toNPs and also to elucidate the interactions of photosensitizing NPs with biological systems. Furthermore, photosensitizing NPs will continue to be designed aiming at targeting organelles since the type of generated ROS is also dependent on where the photosensitizer is located. Other noteworthy field of active research related with NPs is the characterization of biological interactions of NPs. Many researchers have demonstrated that NPs do not reach the target tissue, cell or organelle as they have been designed. Once in the biological medium, NP may associate with biomolecules, like protein, polissacharides and lipids, modifying their “chemical identity” and assuming a new identity, the “biological identity” (Albanese et al., 2014). The new biological identity may change the interaction between NP and cell membrane, changing NPs targeting properties and cytolocalization, which in turn may compromise NP therapeutic efficacy. In this way, the research on NPs for biomedical applications will evolve in the near future to the development of new methods of NPs synthesis and NPs surface modification techniques in order to enhance NP tumor target and controlled intracellular fate. The advances in NP designing coupled with the understanding of chemical and biomolecular interactions between NPs and biological systems are heading to high efficient NP-based drugs for biomedical applications, especially for PDT.

## 6. Experimental design

In this section, we describe selected protocols for NPs preparation and analytical methods mentioned in the previous sections. All the methodologies are described according to the cited references.

### 6.1. Synthesis of photosensitizing NPs

#### 6.1.1. Synthesis of photosensitizing NPs with entraped PS

This approach was used in our previous work where MB or Th was entrapped in silica NPs (Tada et al., 2010). Firstly, 4.2 mL of tetraethylorthosilicate (TEOS) were added to an ethanolic solution of PS (MB or Th) (120 mL; 0.48 mM) and 6.0 mL of ammonia (35%). The mixture was stirred for 24 h at room temperature. The resultant NPs were centrifuged (15000 rpm, 15 min, 25°C) and washed with water and ethanol until no more PS was detected in the supernatant. This method enables high incorporation of PS in the NP but depending on the chosen PS, leakage of PS from NP may be observed even after several washes of photosensitizing NPs.

#### 6.1.2. Attachment of PS to the NP surface

Covalent attachment of PS to NP surface was reported in our previous work (Tada et al., 2010). Thionin was linked to the Cab-O-Sil NP surface (NP of silicon dioxide, Makeni Chemicals Comércio de Produtos Químicos Ltda.). Firstly, 4 g of Cab-O-Sil silica NPs were dried for 8 h at 150°C and then refluxed with 1.5 mL of aminopropyltriethoxysilane (APTS) in dry toluene under stirring for 6 h. The NPs were centrifuged (15000 rpm; 15 min; 25°C) and washed once with dry toluene. After evaporation under reduced pressure the NPs were maintained under vacuum for 12 h at 150°C. Glutaraldehyde (0.5 mL) was then added to 150 mL of NPs suspended in dry DMSO and the mixture was stirred for 40 min at 78°C. The NPs were centrifuged (15000 rpm, 15 min, 25°C) and added to 100 mL of thionin solution in dry DMSO (1.2 mM). The mixture was stirred for 40 min at 78°C. The NPs were centrifuged (15000 rpm, 15 min, 25°C) and washed three times with dry DMSO. The NPs were separated by evaporation under reduced pressure. To reduce the C-N double bonds, 100 mL of dry methanol were added to the NPs and the suspension was refluxed with 4.0 g of NaBH_4_ for 30 min under stirring. After evaporation under reduced pressure the NPs were washed with water and ethanol until no more Th was detected in the supernatant.

Khaing Oo et al. (2012) presented a different method in order to attach PS to gold NP surface. Previously, HAuCl_4_ and branched polyethyleneimine (BPEI) (molecular mass = 10 000) were mixed at various concentrations. The solution mixture was stirred for 5 min in an ice bath and subsequently placed under a metal halide UV lamp (400 W, Cure Zone 2) for 1 h. Upon completion of the reduction reaction, the color of the solution mixture changed from yellow to dark red. Specifically, equal volumes of 4 mol.L^−1^ of HAuCl_4_ and BPEI concentration of 2, 1.4 and 1.2 mg.mL^−1^ were mixed to produce positively charged gold NPs of ~19, ~66, and ~106 nm in diameter, respectively. Gold NPs colloidal solutions (50 μL) of various particle sizes were mixed with 50 μL of 10 μmol.L^−1^ of PpIX solution.

Comparing to the NPs with entrapped PS, PS attachment to the surface has the advantage of no PS leakage from the NP. However, PS loading is generally much lower. Besides, generated ROS are readily delivered to the external medium, reducing the chances of deactivation of excited species inside the NP.

#### 6.1.3. Synthesis of photosensitizing NPs with conjugated PS

In this approach, PS is covalently attached to the molecule of the precursor used to prepare ceramic or polymeric NPs. Differently of the protocols described above, the following examples present the methods used to prepare photosensitizing NPs where PS is present not only embedded into the NPs or only linked to the surface. In the following photosensitizing NPs PS is present into the NP and at the NP surface by covalent bond.

In the work of Rossi et al. (2008), PpIX molecule was modified with a silyl group resulting in the molecule of Silyl-Functionalized Pp IX. First, 100 mg of Pp IX (1.65 × 10^−4^ mol) were refluxed under a dry nitrogen atmosphere with an excess of freshly distilled oxalyl chloride (2 mL). The deep purple solution obtained was refluxed under stirring for 30 min, followed by evaporation of the excess of oxalyl chloride to give Pp IX dichloride as a purple film. The purple residue was stirred with an excess of freshly distilled APTES (1 mL) for 2 h, under a dry nitrogen atmosphere to give dimethyl-8,13-divinyl-3,7,12,17-tetramethyl-21H,23H-porphine-2,18-dipropyl-amidepropyltriethoxysilane(Silyl-Functionalized Pp IX). The product was purified by distillation of the excess APTES and characterized by IR and NMR. Pp IX-Loaded Silica NPs were prepared by adding 200 μL of silyl-functionalized PpIX to an ethanolic solution (100 mL) of ammonia (35%; 6 mL) and TEOS (4.2 mL) under stirring. The mixture was stirred overnight. The NPs were isolated by centrifugation (7000 rpm, 10 min) and washed three times with ethanol, two times with water, and again with ethanol.

Hah et al. (2011) presented the synthesis of photosensitizing NPs by conjugation of MB to PAA molecule. A monomer solution was prepared by dissolving monomers, acrylamide (AA; 610 mg) and N-(3-aminopropyl)methacrylamide hydrochloride (APMA; 45 mg), in PBS (1.4 mL, pH 7.4). An MB succinimidyl ester solution (120 μL; 50 μg.μL^−1^ in DMSO) was added to the monomer solution and the mixture solution was stirred for 2 h at 37°C. 225 mL of 3-(acryloyloxy)-2-hydroxypropylmethacrylate (AHM) were added to the mixture solution under stirring. The MB-containing monomer solution was added to a deoxygenated hexane (36 mL) that consisted of two surfactants, AOT (1.3 g) and Brij 30 (3.2 mL). After stirring the mixture under inert atmosphere for 20 min, a freshly prepared ammonium persulfate solution (10% w/v, 100 mL) and TEMED (100 mL) were added to the mixture solution to initiate polymerization. The solution was then stirred under inert atmosphere at room temperature for 2 h. After completing the polymerization, hexane was removed with a rotary evaporator and the residue was made to a suspension by adding ethanol. The suspension was set in a washing procedure with an Amicon filtration system (Millipore) using a filter membrane (300 kDa) under pressure (1020 psi). The washing procedure was carried out with ethanol five times and with distilled water five times, during which surfactants and unreacted molecules were removed from the product. The resultant MB-conjugated nanoparticles were obtained through a freezedrying.

Comparing to the two other types of NPs described above, these NPs present the advantage of high PS loading and no PS leakage from the NP. Although, due to the higher number of reaction steps this method may result in low final yield.

#### 6.1.4. Synthesis of gold photosensitizing NPs with PS at different distances from the surface

Schneider et al. (2006) reported on the synthesis of photosensitizing NPs by using polymer coated gold NPs. Since this method consists in covalently attaching a PS to a polymer and coating NPs surface with the polymer-PS molecule, this method may be considered as a combination of the conjugated and the attachment of PS to the surface methods, described above. Thus, the resultant NPs present no leakage of PS from NP and also higher ROS availability to the external medium. Firstly, gold NPs were prepared by using the reduction of tetrachloroauric(III) acid (HAuCl_4_.3H_2_O) with trisodium citrate. 70 mL of 38.8× 10^−3^ mol.L^−1^ sodium citrate solution and 700 mL of 10^−1^ mol.L^−1^ HAuCl_4_.3H_2_O solution previously brought to a rolling boil with vigorous stirring. After the synthesis, gold NPs were kept in the dark at 5°C. Following, gold NPs were coated with 1, 5, and 10 layers of poly(allylaminehydrochloride) (PAH; MM = 15000 g.mol^−1^) and poly (styrene sulfonate) (PSS; MM = 13400 g.mol^−1^). For layer deposition several aliquots of the gold NPs suspension were centrifuged for 3 h at 7000 rpm. The supernatant was removed and replaced by the same volume of Milli-Q water. In a typical batch 18 mL of this suspension were added drop per drop, under vigorous stirring, in 18 mL Milli-Q water containing 100 mg of PAH previously sonified in an ultrasonic bath during a few seconds and stirred for 2 h. The agitation was reduced and the mixture kept under stirring at room temperature in the dark during 12 h. These 36 mL were centrifuged during 1h 40 min at 13000 rpm. The supernatant was removed and NPs re-dispersed in Milli-Q water. This centrifugation process was repeated one more time. The total re-dispersion volume after this second centrifugation is adapted in order to obtain a final volume of 18 mL. Then PAH coated gold NPs were added drop per drop, under a vigorous stirring, to 18 mL of a solution of Milli-Q water containing 100 mg of PSS previously sonified in an ultrasonic bath during a few seconds and stirred for 2 h. The agitation was reduced and the mixture kept under stirring at room temperature in the dark during about 12 h and then re-centrifuged. This procedure was followed in cycles to obtain 2, 10, and 20 consecutive polyelectrolyte layers. After 2, 10, and 20 layers deposited, PSS-terminated NPs were concentrated after the 2 centrifugation steps by collecting all liquid pellets in one Eppendorf Safe Lock Tube. The concentration of these concentrated NP suspensions was determined by measuring this absorbance at 438 nm considering that an absorbance of 0.489 corresponds to a NPs concentration of 3 nmol.L^−1^.

### 6.2. Analytical methods

#### 6.2.1. Singlet oxygen generation efficiency

The generation of ^1^O_2_ can be directly and indirectly quantified. In this section we describe two methods of ^1^O_2_ detection and calculation of ^1^O_2_ generation efficiency. The results obtained by direct and indirect methods are generally very similar. Although direct method is the most unambiguous method, it requires a more sophisticated experimental apparatus. Conversely, indirect method requires probes that are commercially available being the main method routinely used in laboratories. In both methods the light scattering by NPs commits the data analysis and may be subtracted from the obtained spectra.

##### 6.2.1.1. Direct method

The singlet oxygen generation efficiency (S) of NPs can be estimated from the relation given in Equation (1), using MB or Th solution in acetonitrile as the standard (Tada et al., 2010).

(1) $$S_{NP}=S_{standard}*\frac{I_{NP}}{I_{standard}}$$

Where: *I_standard_* = phosphorescence emission intensity of ^1^O_2_ at 1270 nm generated by free MB or Th in acetonitrile solution 500 ns after the laser pulse, I_*NP*_ = phosphorescence emission intensity of ^1^O_2_ at 1270 nm generated by NPs in acetonitrile 500 ns after the laser pulse, and *S_standard_* = singlet oxygen quantum yield of free MB or Th in acetonitrile solution given as 0.5 and 0.6, respectively (Bonacin et al., 2009). The absorption values for the standard solutions and particle suspensions were normalized to give the same values of absorption factor at 532 nm. The absorption of the photosensitizer immobilized in the particles was obtained by measuring the absorption spectrum of NPs suspension and subtracting the baseline scattering by multiple point level baseline correction.

##### 6.2.1.2. Indirect method

Herein we described two methodologies based on different probes to monitor ^1^O_2_ generation.

##### 6.2.1.3. Singlet Oxygen Sensor Green (SOSG)

Yoon et al. (2014) used the probe SOSG to detect ^1^O_2_ produced by photosensitizing NPs. A 10 μL aliquot of SOSG (0.5 × 10^−3^ mol.L^−1^ in methanol) was added to a 2 mL of NPs suspension (1 mg.mL^−1^ in PBS (pH 7.4), under constant stirring at 25°C. A “blank spectrum” of SOSG fluorescence (in the absence of ^1^O_2_) was taken by irradiating at 504 nm. After taking a blank spectrum, the photosensitizer sample was irradiated for 5 min at 660 nm. The enhanced SOSG fluorescence spectrum (ex = 504 nm) was obtained immediately after stopping the irradiation of photosensitizer. The arbitrarily defined “S value” constant, for singlet oxygen production, was obtained by calculating the ratio of SOSG fluorescence comparing fluorescence before and after irradiation (enhanced/unenhanced).

##### 6.2.1.4. 1,3-diphenylisobenzofuran (DPBF)

DBPF was used to determine the release of ^1^O_2_ to the solution by photosensitizing NPs in our previous work (Rossi et al., 2008; Tada et al., 2007, 2010). DPBF reacts irreversible with ^1^O_2_, which causes a decrease in the intensity of the DPBF absorption band at around 400 nm. The samples were immediately prepared before use by transferring to a quartz cuvette, in the dark, 40 μL of DBPF stock solution (8 mM) to 2 mL of NP suspension or PS solution at 16°C, so that there was always the same absorption factor at 532 nm (laser wavelength), taking into account the scattering by the solid NPs. The experiments were carried out by irradiating samples with the Nd:YAG 532 nm laser beam while absorption spectra were obtained at certain time intervals. It is important to emphasize the absence of a direct reaction between DPBF and PS (free or in the NP) because the PS absorption of all samples remained unchanged during the experiment. Absorbance at 410 nm was plotted as a function of irradiation time. Under conditions of excess DPBF, pseudo-first order kinetics is observed and the decay time of the DBPF absorption was calculated by applying first-order exponential fitting. The decay time of DPBF (t) is inversely proportional to its reaction rate with ^1^O_2_ which, under these experimental conditions, is proportional to the amount of ^1^O_2_ generated. Considering that the same amount of photons is absorbed by the standards (MB and Th) and samples (photosensitizing NPs), the ^1^O_2_ generation efficiency of the NPs (SNP) was estimated by using Equation (2).

(2) $$S_{NP}=S_{standard}*\frac{t_{NP}}{t_{standard}}$$

Where *t_standard_* = time for the decrease in absorption of DPBF in the presence of MB or Th in acetonitrile solution adjusted to a first-order exponential decay, *t_NP_* = time for the decrease in absorption of DPBF in the presence of NPs in acetonitrile adjusted to a first-order exponential decay and Sstandard is ^1^O_2_ quantum yield of free MB or Th in acetonitrile solution, that is, 0.5 or 0.6, respectively (Bonacin et al., 2009).

#### 6.2.2. Total ROS generation efficiency

The measurement of total ROS generation efficiency is a very convenient method to evaluate photosensitizing NPs photoreactivity, specially when the final application does not require the distinction between ^1^O_2_ and radicals.

Yoon et al. (2014) used the probe anthracence-9,10-dipropionic acid (ADPA) in order to quantify the ROS generated by photosensitizing NPs. A 2mL of photosensitzing NPs suspension (1 mg.mL^−1^ in PBS; pH7.4), containing 80 μL of ADPA (100 μM in pure water), was irradiated at 660 nm, over different time periods (0, 60, 120, 180, 240, 300, 480, and 660 s), under constant stirring at 25°C. The fluorescence spectra of ADPA, excited at 370 nm, were taken right after each irradiation time period. The ADPA decay constant, the “*k* value,” was calculated by the Equation (3) considering that [ROS] is independent of [ADPA] and that [ADPA] decrease only as a consequence of its reaction with generated ROS (Tang et al., 2005).

(3) $$\ln\frac{{\left[ADPA\right]}_{t}}{{\left[ADPA\right]}_{0}}=-kt$$

Where [*ADPA*]_*t*_ = concentration of ADPA at any time *t* and [*ADPA*] = concentration of ADPA at initial time. The value of *k* was extrapolated by a linear fit using the experimental points.

#### 6.2.3. Evaluation of photoactivity

Photosensitizing NPs photoactivity may be evaluated by measuring cell viability of cells after PDT treatment with the NPs. MTT assay and fluorescence staining are two of the most applied methods to measure cell viability. The first one has been reported as a low accuracy method in the sense that it measures the mitochondrial activity of cells which can be high even if the cells are already damaged by the photoinduced process. However, it is a very convenient method that does not require fluorescent probes and a fluorescence microscope, as it is required by the immunofluorescent-staining method, which may endear the experimental protocol.

##### 6.2.3.1. Cell viability measurement by MTT assay

In order to evaluate photoactivity of photosensitizing NPs, Khaing Oo et al. (2012) and Li et al. (2013) used the thiazolyl blue tetrazolium bromide (MTT) assay. In Li et al. (2013), adenocarcinomic human alveolar basal epithelial (A549) cells were cultured at 37°C in RPMI-1640 containing 10% fetal calf serum under a humidified atmosphere containing 5% of CO_2_. Cells were seeded in 96-well plates at a density of 1 × 10^4^ viable cells per well and pre-incubated for 24 h to allow cell attachment. The cells were then incubated with free PS and photosensitizing NPs. After 12 h of incubation, the medium was replaced with fresh RPMI 1640 just before irradiation at 1.5 J/cm^2^ by a 670 nm laser source. After 24 h of incubation, viability of cells after treatment and viability of cells kept in the dark were determined by MTT assay. As described by Khaing Oo et al. (2012), the culture was incubated with MTT in cell culture medium (0.5 mg/mL) for 2 h. After discarding the nonreacted solution, 100 μL of DMSO was added to extract the formazan crystals. Absorbance of the extract was measured at 570 nm with a microplate reader. The experiment was repeated at least three times.

##### 6.2.3.2. Immunofluorescent-staining

Yang et al. (2014) used a Live/Dead Viability/Cytotoxicity kit (Invitrogen, Carlsbad, CA) in order to analyze cell viability after treatment with photosensitizing NPs (Yang et al., 2014). MDA-MB-231 cells after various PDT treatments were fluorescently stained for viability using the immunofluorescent kit according to the manufacturer protocol. Briefly, cells cultured on glass cover slips after various PDT treatments were washed with HBSS and incubated with 2 μM calcein acetoxymethyl (Calcein AM, 0.05%) and 0.5 μM ethidium homodimer-1 (EthD-1, 0.2%) in HBSS for 30 min in the incubator. Viable cells were stained fluorescent green by Calcein AM, while the nuclei of dead cells were stained fluorescent red by EthD-1 in case of membranolysis. The stained cells were examined under a Nikon Eclipse 80i epi-fluorescence microscope.

### Conflict of interest statement

The authors declare that the research was conducted in the absence of any commercial or financial relationships that could be construed as a potential conflict of interest.

## References

[B1] Albanese A. Walkey C. D. Olsen J. B. Guo H. Emili A. Chan W. C. W. (2014). Secreted biomolecules alter the biological identity and cellular interactions of nanoparticles. ACS Nano 8, 5515–5526. 10.1021/nn4061012 24797313

[B2] Almeida R. D. Manadas B. J. Carvalho A. P. Duarte C. B. (2004). Intracellular signaling mechanisms in photodynamic therapy. Biochim. Biophys. Acta 1704, 59–86. 10.1016/j.bbcan.2004.05.003 15363861

[B3] An B.-K. Gierschner J. Park S. Y. (2012). Ï-conjugated cyanostilbene derivatives: a unique self-assembly motif for molecular nanostructures with enhanced emission and transport. Acc. Chem. Res. 45, 544–554. 10.1021/ar2001952 22085759

[B4] Baba K. Pudavar H. E. Roy I. Ohulchanskyy T. Y. Chen Y. Pandey R. K.. (2007). New method for delivering a hydrophobic drug for photodynamic therapy using pure nanocrystal form of the drug. Mol. Pharm. 4, 289–297. 10.1021/mp060117f 17266331 PMC2667689

[B5] Bonacin J. A. Engelmann F. A. M. Severino D. Toma H. E. Baptista M. S. (2009). Singlet oxygen quantum yields in water using beetroot extract and an array of leds. J. Braz. Chem. Soc. 20, 31–36. 10.1590/S0103-50532009000100006

[B6] Brown S. B. Brown E. A. Walker I. (2004). The present and future role of photodynamic therapy in cancer treatment. Lancet Oncol. 5, 497–508. 10.1016/S1470-2045(04)01529-3 15288239

[B7] Castano A. P. Demidova T. N. Hamblin M. R. (2004). Mechanisms in photodynamic therapy: part oneâphotosensitizers, photochemistry and cellular localization. Photodiagnosis Photodyn. Ther. 1, 279–293. 10.1016/S1572-1000(05)00007-4 25048432 PMC4108220

[B8] Chen L. L. Wang S. Q. (2012). From the bottle to the skin: challenges in evaluating antioxidants. Photodermatol. Photoimmunol. Photomed. 28, 228–234. 10.1111/j.1600-0781.2012.00674.x 22971186

[B9] Cheng Y. Samia A. C. Meyers J. D. Panagopoulos I. Fei B. Burda C. (2008). Highly efficient drug delivery with gold nanoparticle vectors for *in vivo* photodynamic therapy of cancer. J. Am. Chem. Soc. 130, 10643–10647. 10.1021/ja801631c 18642918 PMC2719258

[B10] Cheng Y. Meyers J. D. Broome A.-M. Kenney M. E. Basilion J. P. Burda C. (2011). Deep penetration of a pdt drug into tumors by noncovalent drug-gold nanoparticle conjugates. J. Am. Chem. Soc. 133, 2583–2591. 10.1021/ja108846h 21294543 PMC3056176

[B11] Chiarelli-Neto O. Ferreira A. S. Martins W. K. Pavani C. Severino D. Faião-Flores F.. (2014). Melanin photosensitization and the effect of visible light on epithelial cells. PLoS ONE 9:e113266. 10.1371/journal.pone.0113266 25405352 PMC4236153

[B12] Cho K.-H. Park J.-E. Osaka T. Park S.-G. (2005). The study of antimicrobial activity and preservative effects of nanosilver ingredient. Electrochim. Acta 51, 956–960. 10.1016/j.electacta.2005.04.071

[B13] Christensen I. L. Sun Y.-P. Juzenas P. (2011). Carbon dots as antioxidants and prooxidants. J. Biomed. Nanotechnol. 7, 667–676. 10.1166/jbn.2011.1334 22195484

[B14] Cincotta L. Foley J. W. Cincotta A. H. (1993). Phototoxicity, redox behavior, and pharmacokinetics of benzophenoxazine analogues in emt-6 murine sarcoma cells. Cancer Res. 53, 2571–2580. 8495421

[B15] Costa J. C. S. Ando R. A. Camargo P. H. C. Corio P. (2011). Understanding the effect of adsorption geometry over substrate selectivity in the surface-enhanced raman scattering spectra of simazine and atrazine. J. Phys. Chem. C 115, 4184–4190. 10.1021/jp112021j

[B16] de Assis F. F. de Souza J. M. Assis B. H. Brocksom T. J. de Oliveira K. T. (2013). Synthesis and photophysical studies of a chlorin sterically designed toâ prevent self-aggregation. Dyes Pigm. 98, 153–159. 10.1016/j.dyepig.2013.02.011

[B17] Deda D. K. Pavani C. Caritá E. Baptista M. S. Toma H. E. Araki K. (2013). Control of cytolocalization and mechanism of cell death by encapsulation of a photosensitizer. J. Biomed. Nanotechnol. 9, 1307–1317. 10.1166/jbn.2013.1614 23926796

[B18] Detty M. R. Gibson S. L. Wagner S. J. (2004). Current clinical and preclinical photosensitizers for use in photodynamic therapy. J. Med. Chem. 47, 3897–3915. 10.1021/jm040074b 15267226

[B19] Dewaele M. Maes H. Agostinis P. (2010). Ros-mediated mechanisms of autophagy stimulation and their relevance in cancer therapy. Autophagy 6, 838–854. 10.4161/auto.6.7.12113 20505317

[B20] Doane T. L. Burda C. (2012). The unique role of nanoparticles in nanomedicine: imaging, drug delivery and therapy. Chem. Soc. Rev. 41, 2885–2911. 10.1039/c2cs15260f 22286540

[B21] dos Santos F. A. Uchoa A. F. Baptista M. S. Iamamoto Y. Serra O. A. Brocksom T. J. (2013). Synthesis of functionalized chlorins sterically-prevented from self-aggregation. Dyes Pigm. 99, 402–411. 10.1016/j.dyepig.2013.05.024

[B22] Dulkeith E. Morteani A. C. Niedereichholz T. Klar T. A. Feldmann J. Levi S. A.. (2002). Fluorescence quenching of dye molecules near gold nanoparticles: radiative and nonradiative effects. Phys. Rev. Lett. 89:203002. 10.1103/PhysRevLett.89.203002 12443474

[B23] Fan Z. Dai X. Lu Y. Yu E. Brahmbatt N. Carter N.. (2014). Enhancing targeted tumor treatment by near ir light-activatable photodynamicâphotothermal synergistic therapy. Mol. Pharm. 11, 1109–1116. 10.1021/mp4002816 24568338 PMC3983349

[B24] Faraji A. H. Wipf P. (2009). Nanoparticles in cellular drug delivery. Bioorg. Med. Chem. 17, 2950–2962. 10.1016/j.bmc.2009.02.043 19299149

[B25] Farhadi A. Roxin Á. Wilson B. C. Zheng G. (2015). Nano-enabled sers reporting photosensitizers. Theranostics 5, 469–476. 10.7150/thno.10694 25767614 PMC4350009

[B26] Faulds K. Hernandez-Santana A. Smith W. E. (2010). The inorganic chemistry of surface enhanced raman scattering (sers), in Spectroscopic Properties of Inorganic and Organometallic Compounds: Techniques, Materials and Applications, Vol. 41, eds Yarwood J. Douthwaite R. Duckett S. B. (Cambridge, UK: The Royal Society of Chemistry), 1–21.

[B27] Gabrielli D. Belisle E. Severino D. Kowaltowski A. J. Baptista M. S. (2004). Binding, aggregation and photochemical properties of methylene blue in mitochondrial suspensions. Photochem. Photobiol. 79, 227–232. 10.1562/BE-03-27.1 15115294

[B28] Ghosh S. Ucer K. B. D'Agostino R. Jr. Grant K. Sirintrapun J. Thomas M. J.. (2014). Non-covalent assembly of meso-tetra-4-pyridyl porphine with single-stranded dna to form nano-sized complexes with hydrophobicity-dependent dna release and anti-tumor activity. Nanomedicine 10, 451–461. 10.1016/j.nano.2013.07.019 23988714 PMC3946208

[B29] Hah H. J. Kim G. Lee Y.-E. K. Orringer D. A. Sagher O. Philbert M. A.. (2011). Methylene blue-conjugated hydrogel nanoparticles and tumor-cell targeted photodynamic therapy. Macromol. Biosci. 11, 90–99. 10.1002/mabi.201000231 20976722

[B30] Hamblin M. R. Hasan T. (2004). Photodynamic therapy: a new antimicrobial approach to infectious disease? Photochem. Photobiol. Sci. 3, 436–450. 10.1039/b311900a 15122361 PMC3071049

[B31] Herrling T. Jung K. Fuchs J. (2006). Measurements of uv-generated free radicals/reactive oxygen species (ros) in skin. Spectrochim. Acta A Mol. Biomol. Spectrosc. 63, 840–845. 10.1016/j.saa.2005.10.013 16543118

[B32] Hilf R. (2007). Mitochondria are targets of photodynamic therapy. J. Bioenerg. Biomembr. 39, 85–89. 10.1007/s10863-006-9064-8 17334915

[B33] Ichinose S. Usuda J. Hirata T. Inoue T. Ohtani K. Maehara S.. (2006). Lysosomal cathepsin initiates apoptosis, which is regulated by photodamage to bcl-2 at mitochondria in photodynamic therapy using a novel photosensitizer, atx-s10 (na). Int. J. Oncol. 29, 349–355. 10.3892/ijo.29.2.349 16820876

[B34] Junqueira H. C. Severino D. Dias L. G. Gugliotti M. S. Baptista M. S. (2002). Modulation of methylene blue photochemical properties based on adsorption at aqueous micelle interfaces. Phys. Chem. Chem. Phys. 4, 2320–2328. 10.1039/b109753a

[B35] Kang K. Wang J. Jasinski J. Achilefu S. (2011). Fluorescence manipulation by gold nanoparticles: from complete quenching to extensive enhancement. J. Nanobiotechnol. 9:16 10.1186/1477-3155-9-16 PMC3112388 21569249

[B36] Khaing Oo M. K. Yang Y. Hu Y. Gomez M. Du H. Wang H. (2012). Gold nanoparticle-enhanced and size-dependent generation of reactive oxygen species from protoporphyrin ix. ACS Nano 6, 1939–1947. 10.1021/nn300327c 22385214

[B37] Kochevar I. E. Lynch M. C. Zhuang S. Lambert C. R. (2000). Singlet oxygen, but not oxidizing radicals, induces apoptosis in hl-60 cellsâ. Photochem. Photobiol. 72, 548–553. 10.1562/0031-8655(2000)072<0548:SOBNOR>2.0.CO;2 11045728

[B38] Konan Y. N. Gurny R. Allémann E. (2002). State of the art in the delivery of photosensitizers for photodynamic therapy. J. Photochem. Photobiol. B 66, 89–106. 10.1016/S1011-1344(01)00267-6 11897509

[B39] Kumar R. Roy I. Ohulchanskyy T. Y. Goswami L. N. Bonoiu A. C. Bergey E. J.. (2008). Covalently dye-linked, surface-controlled, and bioconjugated organically modified silica nanoparticles as targeted probes for optical imaging. ACS Nano 2, 449–456. 10.1021/nn700370b 19206569

[B40] Li L. Nurunnabi M. Nafiujjaman M. Lee Y. K. Huh K. M. (2013). Gsh-mediated photoactivity of pheophorbide a-conjugated heparin/gold nanoparticle for photodynamic therapy. J. Control. Release 171, 241–250. 10.1016/j.jconrel.2013.07.002 23867285

[B41] Lkhagvadulam B. Kim J. H. Yoon I. Shim Y. K. (2013). Size-dependent photodynamic activity of gold nanoparticles conjugate of water soluble purpurin-18-n-methyl-d-glucamine. Biomed. Res. Int. 2013:720579 10.1155/2013/720579 23533998 PMC3591214

[B42] Macdonald I. J. Dougherty T. J. (2001). Basic principles of photodynamic therapy. J. Porphyr. Phthalocyanines 5, 105–129. 10.1002/jpp.328

[B43] Managa M. Antunes E. Nyokong T. (2014). Conjugates of platinum nanoparticles with gallium tetra â (4-carboxyphenyl) porphyrin and their use in photodynamic antimicrobial chemotherapy when in solution or embedded in electrospun fiber. Polyhedron 76, 94–101. 10.1016/j.poly.2014.03.050

[B44] Morgan J. Oseroff A. R. (2001). Mitochondria-based photodynamic anti-cancer therapy. Adv. Drug Deliv. Rev. 49, 71–86. 10.1016/S0169-409X(01)00126-0 11377804

[B45] Narband N. Tubby S. Parkin I. P. Gil-Tomas J. Ready D. Nair S. P. (2008). Gold nanoparticles enhance the toluidine blue-induced lethal photosensitisation of staphylococcus aureus. Curr. Nanosci. 4, 409–414. 10.2174/157341308786306134

[B46] Nogueira A. E. Longo E. Leite E. R. Camargo E. R. (2014). Synthesis and photocatalytic properties of bismuth titanate with different structures via oxidant peroxo method (opm). J. Colloid Interface Sci. 415, 89–94. 10.1016/j.jcis.2013.10.010 24267334

[B47] Oleinick N. L. Morris R. L. Belichenko I. (2002). The role of apoptosis in response to photodynamic therapy: what, where, why, and how. Photochem. Photobiol. Sci. 1, 1–21. 10.1039/b108586g 12659143

[B48] Oliveira C. S. Turchiello R. Kowaltowski A. J. Indig G. L. Baptista M. S. (2011). Major determinants of photoinduced cell death: subcellular localization versus photosensitization efficiency. Free Radic. Biol. Med. 51, 824–833. 10.1016/j.freeradbiomed.2011.05.023 21664269

[B49] Park J. Lee J. Choi C. (2011). Mitochondrial network determines intracellular ros dynamics and sensitivity to oxidative stress through switching inter-mitochondrial messengers. PLoS ONE 6:e23211. 10.1371/journal.pone.0023211 21829717 PMC3150422

[B50] Pelletier J. Transue S. Snyder E. (2006). Pathogen inactivation techniques. Best Pract. Res. Clin. Haematol. 19, 205–242. 10.1016/j.beha.2005.04.001 16377551 PMC7106341

[B51] Rossi L. M. Silva P. R. Vono L. L. R. Fernandes A. U. Tada D. B. Baptista M. S. (2008). Protoporphyrin ix nanoparticle carrier: Preparation, optical properties, and singlet oxygen generation. Langmuir 24, 12534–12538. 10.1021/la800840k 18834155

[B52] Samia A. C. S. Chen X. Burda C. (2003). Semiconductor quantum dots for photodynamic therapy. J. Am. Chem. Soc. 125, 15736–15737. 10.1021/ja0386905 14677951

[B53] Samia A. C. S. Dayal S. Burda C. (2006). Quantum dot-based energy transfer: Perspectives and potential for applications in photodynamic therapy. Photochem. Photobiol. 82, 617–625. 10.1562/2005-05-11-IR-525 16475871

[B54] Schneider G. Decher G. Nerambourg N. Praho R. Werts M. H. V. Blanchard-Desce M. (2006). Distance-dependent fluorescence quenching on gold nanoparticles ensheathed with layer-by-layer assembled polyelectrolytes. Nano Lett. 6, 530–536. 10.1021/nl052441s 16522057

[B55] Severino D. Junqueira H. C. Gugliotti M. Gabrielli D. S. Baptista M. S. (2003). Influence of negatively charged interfaces on the ground and excited state properties of methylene blue. Photochem. Photobiol. 77, 459–468. 10.1562/0031-8655(2003)077<0459:IONCIO>2.0.CO;2 12812286

[B56] Tada D. B. Rossi L. M. Leite C. A. P. Itri R. Baptista M. S. (2010). Nanoparticle platform to modulate reaction mechanism of phenothiazine photosensitizers. J. Nanosci. Nanotechnol. 10, 3100–3108. 10.1166/jnn.2010.2165 20358905

[B57] Tada D. B. Suraniti E. Rossi L. M. Leite C. A. P. Oliveira C. S. Tumolo T. C.. (2014). Effect of lipid coating on the interaction between silica nanoparticles and membranes. J. Biomed. Nanotechnol. 10, 519–528. 10.1166/jbn.2014.1723 24730247

[B58] Tada D. B. Vono L. L. R. Duarte E. L. Itri R. Kiyohara P. K. Baptista M. S.. (2007). Methylene blue-containing silica-coated magnetic particles: a potential magnetic carrier for photodynamic therapy. Langmuir 23, 8194–8199. 10.1021/la700883y 17590032

[B59] Tam N. C. M. McVeigh P. Z. MacDonald T. D. Farhadi A. Wilson B. C. Zheng G. (2012). Porphyrin–lipid stabilized gold nanoparticles for surface enhanced raman scattering based imaging. Bioconjug. Chem. 23, 1726–1730. 10.1021/bc300214z 22876736

[B60] Tang W. Xu H. Kopelman R. Philbert M. A. (2005). Photodynamic characterization and *in vitro* application of methylene blue-containing nanoparticle platforms. Photochem. Photobiol. 81, 242–249 10.1562/2004-05-24-RA-176.1 15595888

[B61] Tardivo J. P. Giglio A. D. de Oliveira C. S. Gabrielli D. S. Junqueira H. C. Tada D. B.. (2005). Methylene blue in photodynamic therapy: from basic mechanisms to clinical applications. Photodiagnosis Photodyn. Ther. 2, 175–191. 10.1016/S1572-1000(05)00097-9 25048768

[B62] Uchoa A. F. de Oliveira K. T. Baptista M. S. Bortoluzzi A. J. Iamamoto Y. Serra O. A. (2011). Chlorin photosensitizers sterically designed to prevent self-aggregation. J. Org. Chem. 76, 8824–8832. 10.1021/jo201568n 21932835

[B63] Wang J. Yi J. (2008). Cancer cell killing via ros: to increase or decrease, that is the question. Cancer Biol. Ther. 7, 1875–1884. 10.4161/cbt.7.12.7067 18981733

[B64] Wu W.-S. (2006). The signaling mechanism of ros in tumor progression. Cancer Metastasis Rev. 25, 695–705. 10.1007/s10555-006-9037-8 17160708

[B65] Yang Y. Hu Y. Du H. Wang H. (2014). Intracellular gold nanoparticle aggregation and their potential applications in photodynamic therapy. Chem. Commun. 50, 7287–7290. 10.1039/c4cc02376e 24871860

[B66] Yao M. Gu C. Doyle F. J. Zhu H. Redmond R. W. Kochevar I. E. (2014). Why is rose bengal more phototoxic to fibroblasts *in vitro* than *in vivo*? Photochem. Photobiol. 90, 297–305. 10.1111/php.12215 24266530

[B67] Yoon H. K. Lou X. Chen Y.-C. Koo Lee Y.-E. Yoon E. Kopelman R. (2014). Nanophotosensitizers engineered to generate a tunable mix of reactive oxygen species, for optimizing photodynamic therapy, using a microfluidic device. Chem. Mater. 26, 1592–1600. 10.1021/cm403505s 24701030 PMC3970790

[B68] Zhao Y. Ye C. Liu W. Chen R. Jiang X. (2014). Tuning the composition of aupt bimetallic nanoparticles for antibacterial application. Angew. Chem. Int. Ed. 53, 8127–8131. 10.1002/anie.201401035 24828967 PMC4320751

